# A novel small-molecule activator of Sirtuin-1 induces autophagic cell death/mitophagy as a potential therapeutic strategy in glioblastoma

**DOI:** 10.1038/s41419-018-0799-z

**Published:** 2018-07-10

**Authors:** Zhi-qiang Yao, Xin Zhang, Yongqi Zhen, Xu-Ying He, Shuangmei Zhao, Xi-Feng Li, Bo Yang, Feng Gao, Fu-You Guo, Leilei Fu, Xian-Zhi Liu, Chuan-Zhi Duan

**Affiliations:** 1 The National Key Clinical Specialty, The Engineering Technology Research Center of Education of China, Guangdong Provincial Key Laboratory on Brain Function Repair and Regeneration, Department of Neurosurgery, Zhujiang Hospital, Southern Medical University, Guangzhou, 510282 China; 2 Department of Interventional Neuroradiology, The First Affiliated Hospital of Zhengzhou University, 1 Jianshe Road, Zhengzhou, 450052 China; 3 School of Life Science and Engineering, Southwest Jiaotong University, Chengdu, 610031 China; 4 Department of Neurosurgery, The First Affiliated Hospital of Zhengzhou University, 1 Jianshe Road, Zhengzhou, 450052 China

## Abstract

Sirtuin-1 (SIRT1), the mammalian ortholog of yeast Sir2p, is well known to be a highly conserved NAD^+^-dependent protein deacetylase that has been emerging as a key cancer target. Autophagy, an evolutionarily conserved, multi-step lysosomal degradation process, has been implicated in cancer. Accumulating evidence has recently revealed that SIRT1 may act as a tumor suppressor in several types of cancer, and thus activating SIRT1 would represent a possible therapeutic strategy. Thus, in our study, we identified that SIRT1 was a key prognostic factor in brain cancer based upon The Cancer Genome Atlas and tissue microarray analyses. Subsequently, we screened a series of potential small-molecule activators of SIRT1 from Drugbank, and found the best candidate compound F0911-7667 (hereafter, named Comp **5**), which showed a good deacetylase activity for SIRT1 rather than other Sirtuins. In addition, we demonstrated that Comp **5**-induced autophagic cell death via the AMPK-mTOR-ULK complex in U87MG and T98G cells. Interestingly, Comp **5-**induced mitophagy by the SIRT1–PINK1–Parkin pathway. Further iTRAQ-based proteomics analyses revealed that Comp **5** could induce autophagy/mitophagy by downregulating 14-3-3γ, catalase, profilin-1, and HSP90α. Moreover, we showed that Comp **5** had a therapeutic potential on glioblastoma (GBM) and induced autophagy/mitophagy by activating SIRT1 in vivo. Together, these results demonstrate a novel small-molecule activator of SIRT1 that induces autophagic cell death/mitophagy in GBM cells, which would be utilized to exploit this compound as a leading drug for future cancer therapy.

## Introduction

Sirtuin-1 (SIRT1), a NAD^+^-dependent protein deacetylase, catalyzes the removal of acetyl groups from lysine residues in substrate proteins. It recruits transcriptional machinery to target promoters to induce transcriptional changes^1^. The deacetylating activity of SIRT1 regulates variety of biological processes such as axonal integrity, autophagy and so on^2,3^. And, SIRT1 is highly expressed in several organs like brain and spinal cord^4^. In adult rodent brains, the mRNA of SIRT1 is abundantly expressed in metabolically relevant areas, the hypothalamic arcuate, ventromedial, dorsomedial, paraventricular nuclei, the area postrema and the nucleus of the solitary tract in the hindbrain^5^. Numerous studies of multiple sclerosis (MS) showed that SIRT1 was upregulated in acute and chronic brain lesions, and reduced in the peripheral blood during MS exacerbations, and the overexpression of SIRT1 was neuroprotective^6^. As mentioned above, SIRT1 may function as a key target in neurological diseases.

Autophagy refers to an evolutionarily conserved, multi-step lysosomal degradation process in which the cell degrades long-lived proteins and damaged organelles^7^. Macroautophagy (autophagy) is the major regulated catabolic mechanism that involves the delivery of cytoplasmic cargo, which sequestered inside double-membrane vesicles to the lysosome, highly regulated by a few autophagy-related signaling pathways, such as AMPK-mTOR-ULK1. Recent studies have indicated that autophagy may play a tumor suppressor role, connected to its role in the clearance of the protein p62^8^. However, as autophagy is often regard as a survival mechanism, cancer cells could also exploit it to survive nutrient limitation and hypoxia that often occur in solid tumors^9^. Tumor cells can also upregulate autophagy as a response to cancer therapies, and it was reported that inhibition of autophagy could enhance the killing of tumor cells after treatment^10^. In the past decade, several studies have demonstrated that autophagy-dependent cell death occurs under certain experimental conditions such as excessive cellular stress or treatment with chemotherapeutic agents or other toxic compounds^11,12^. Therefore, targeting autophagy is a significant therapeutic avenue in cancer treatment.

The regulation of SIRT1 has been reported to be involved in the mechanisms of autophagy in many diseases. For instance, overexpression of SIRT1 in neurons could prevent the accumulation of the prion protein and neurotoxicity by inducing autophagy^13^. Fluoride has also been reported to activate SIRT1 phosphorylation and to initiate autophagy, resulting in the protection of ameloblasts cells from the fluoride-induced endoplasmic reticulum stress and eliminating the interruption of enamel formation^14^. It suggested that autophagy induction by the activation of SIRT1 might be a promising therapeutic strategy to ameliorate the development of neurological cancer.

Of note, some achievements have been made in cancer therapies targeting SIRT1 with autophagy mechanisms. However, the applications of “SIRT1-modulating autophagy” in brain cancer remains in its infancy. Thus, in this study, we carried out The Cancer Genome Atlas (TCGA) and tissue microarray (TMA) analyses, in silico drug design and screening, molecular pharmacological technologies and iTRAQ-based proteomics analyses, which together help us discovering a novel small-molecule activator of SIRT1 that induces autophagic cell death/mitophagy in glioblastoma in vitro and in vivo. Thus, these findings would shed light on exploiting this SIRT1 activator as a leading drug for future glioblastoma therapy.

## Results

### Identification of SIRT1 as a prognostic factor in brain cancer

**Fig. 1:**
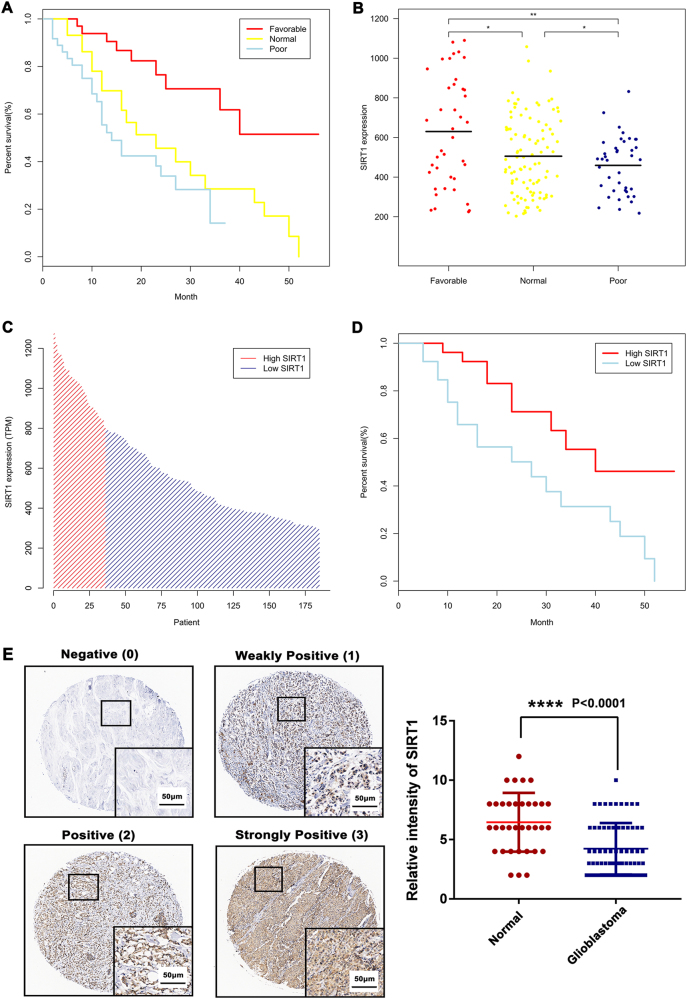
Identification of SIRT1 as a prognostic factor in brain cancer. **a** Cytogenetics risk category is the single most important prognostic factor in brain cancer. **b** One hundred and eighty-three brain cancer patients, from the TCGA data set, were clustered into three groups according to cytogenetics risk category (favorable: *N* = 40; Normal: *N* = 103; Poor: *N* = 40). **c** Patients were clustered into two groups according to the expression levels of SIRT1 (low-SIRT1 expression group: *N* = 36; high-SIRT1 expression group: *N* = 147). **d** Kaplan–Meier analysis reveals a positive correlation between patient overall survival rates and SIRT1 mRNA expression levels. **e** Representative immunoreactivity intensities of SIRT1 for negative (0), weakly positive (1), positive (2), strongly positive (3) staining. Scale bar = 50 μm. The expression of SIRT1 is downregulated in glioblastoma tissues (*P* < 0.0001) compared with normal brain tissues

We investigated the correlation between the expression level of SIRT1 and the clinical prognoses of brain cancer patients. In TCGA data set (183 brain cancer patients with cytogenetics information), the mRNA expression of SIRT1 was associated with cytogenetics risk category of patients, which was one of the most important prognostic factors in brain cancer (Fig. 1a). High level of SIRT1 mRNA expression was strongly associated with the favorable group, compared with the normal group (*P* = 0.0172) and the poor group (*P* = 0.0083) (Fig. 1b). Brain cancer patients were clustered into the high-SIRT1 expression group (*N* = 36) and the low-SIRT1 expression group (*N* = 147) (Fig. 1c). Kaplan–Meier analysis of revealed a positive correlation between the overall survival of patients and the mRNA expression of SIRT1 (*P* = 0.029), indicating the prognostic significance of SIRT1 in brain cancer (Fig. 1d). To further demonstrate that SIRT1 is downregulated in brain cancer, we applied three TMA containing 70 samples of glioblastoma and 35 samples of normal brain tissues to evaluate the expression of SIRT1. Immunohistochemical analyses demonstrated decreased expression of SIRT1 in glioblastoma samples compared with normal brain tissues (*P* < 0.0001) (Fig. 1e). Together, these results indicate that SIRT1 is downregulated in brain cancer, especially in glioblastoma.

### In silico design and screening of potential SIRT1 activators

**Fig. 2:**
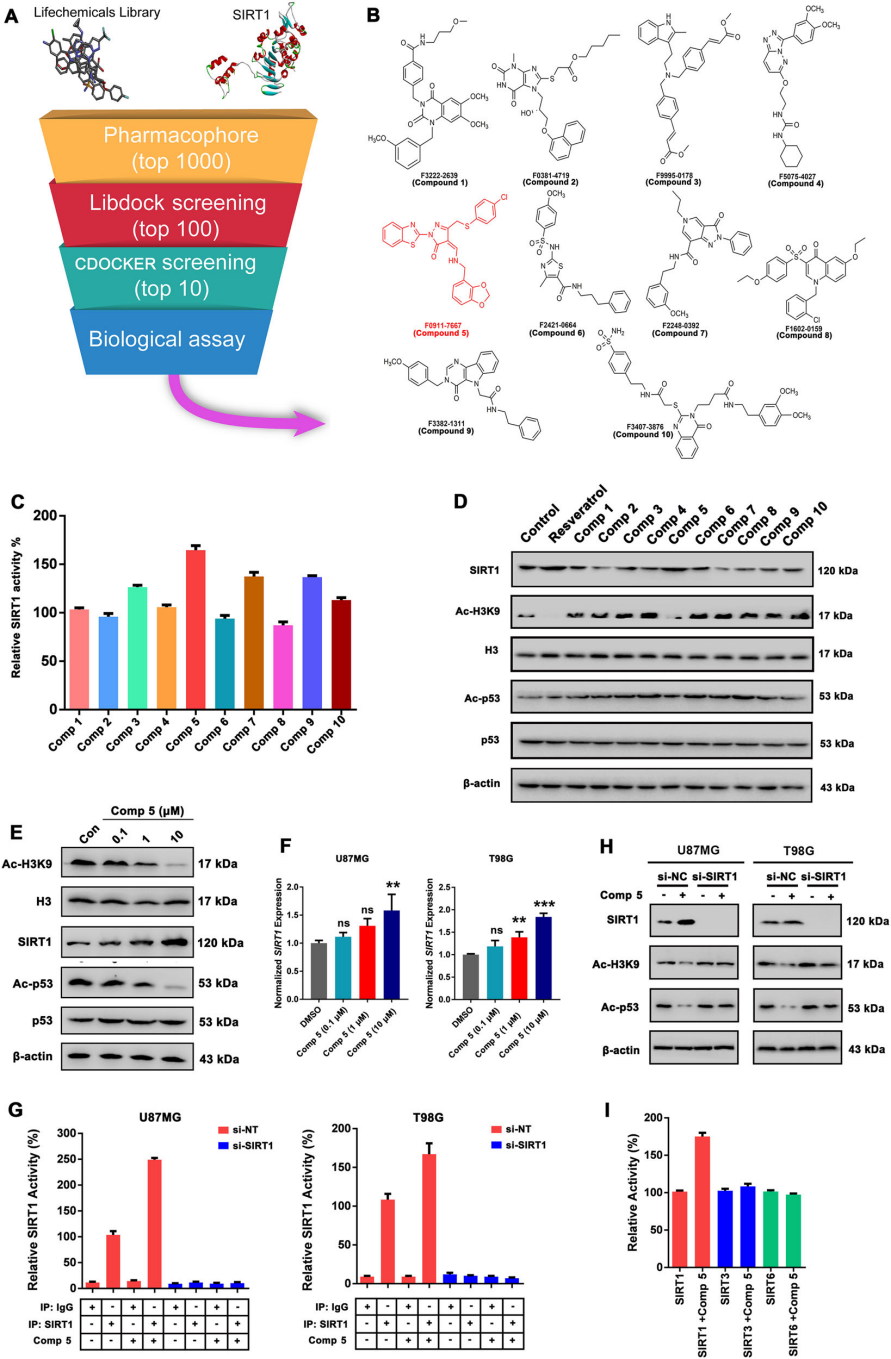
Discovery of novel SIRT1 activators. **a** Virtual screening schematic model for the discovery of novel SIRT1 activators. **b** Chemical structure of the top 10 candidate SIRT1 activators. **c** The top 10 candidate compounds (1 μM) were screened for deacetylase activity by SIRT1 activity assay. **d** U87MG cells were incubated with top 10 candidate compounds (1 μM) for 24 h, then detected by western blot for acetylated or total H3 or SIRT1 protein, as well as the acetylated or total p53. Resveratrol (1 μM) was used as a positive control for SIRT1 activator. **e** U87MG cells were incubated with 0.1, 1, 10 μM Comp **5** for 24 h, then detected by western blot for acetylated or total H3 or SIRT1 protein, as well as the acetylated or total p53. β-actin was used as a loading control. **f** The mRNA expression levels of *SIRT1* in U87MG and T98G cells treated with 0.1, 1, 10 μM Comp **5** were quantified by real-time PCR. The values obtained from the control group were set at 1.0. Values were means ± SD, *n* = 3 per group. ***p < 0.001; **p < 0.01. **g** The cellular SIRT1 deacetylase activities of Comp **5** were determined by SIRT1 activity assay kit using cellular extracts obtained from normal or SIRT1-silenced U87MG and T98G cells. **h** U87MG and T98G cells were transfected with SIRT1 siRNA for indicated time and treated with Comp **5** for additional 24 h. The expression levels of SIRT1 as well as acetylated H3K9 or p53 were determined by western blot. **i** The in vitro SIRTs deacetylase activities of Comp **5** were determined by SIRT1, SIRT3, and SIRT6 activity assay kits

Our virtual screening strategy consisted of using pharmacophore searching and docking methods. At first, we used virtual screening of chemical libraries that based upon the Lifechemicals library, searching for novel leading compounds with distinctive skeleton based on the pharmacophore model constructed from the X-ray structure of SIRT1 (PDB code: 4ZZJ) (Fig. 2a). The top 1000 compounds were screened by the pharmacophore model from Lifechemicals library, and the top 100 hits were selected by LibDock protocol in the first step. Subsequently, top 20 hits were further determined by CDOCKER protocol. Finally, the top 10 hits were reselected according to the results of SIRT1 deacetylase activity assay (Fig. 2b, c). As a result, the compound F0911-7667 (hereafter, refers to Comp **5**) with the most potent SIRT1 activity was selected to the best candidate SIRT1 activator (Fig. 2c). To determine whether the top 10 hits activate SIRT1 in cells, we first checked if SIRT1 activity is indeed elevated in cells treated with these compounds at 1 μM. Similar to the in vitro SIRT1 deacetylase activity results, we found that the acetylation level of histone H3, a known SIRT1 substrate^15^, was markedly reduced after treatments of Comp **5** or resveratrol, indicating an increase of SIRT1 activity (Fig. 2d). And the protein levels of SIRT1 were also increased in the Comp **5**- or resveratrol-treated cells (Fig. 2d). In addition, the p53 acetylation level appeared to be much lower in the presence of Comp **5** or resveratrol, suggesting an activation of SIRT1 upon the treatment of Comp **5** (Fig. 2d). In addition, we also found that Comp **5** could decrease the acetylation of H3K9 and p53, as well as *SIRT1* mRNA expression in dose-dependent manners (Fig. 2e, f). To demonstrate that Comp **5** is an activator of SIRT1, we determined the cellular deacetylase activity of SIRT1 in U87MG and T98G cells after Comp **5** treatment. As a result, Comp **5** significantly increased cellular SIRT1 deacetylase activity (Fig. 2g). Interestingly, Comp **5** did not activate SIRT1 in cells or deacetylate H3K9 and p53 in SIRT1-silenced cells (Fig. 2g, h). Moreover, we also examined the relative activities of other SIRTs such as SIRT3 and SIRT6 and demonstrated that Comp **5** could only increase SIRT1 activity rather than SIRT3 or SIRT6 (Fig. 2i). Based upon above-mentioned results, Comp **5** displayed certain selectivity in different SIRTs deacetylase activities, but it also increase the expression of SIRT1 in protein and mRNA levels. Therefore, we conclude that Comp **5** might be an activator of SIRT1 without specificity.

### Comp 5 induces autophagy via the AMPK-mTOR-ULK complex in GBM cells

**Fig. 3:**
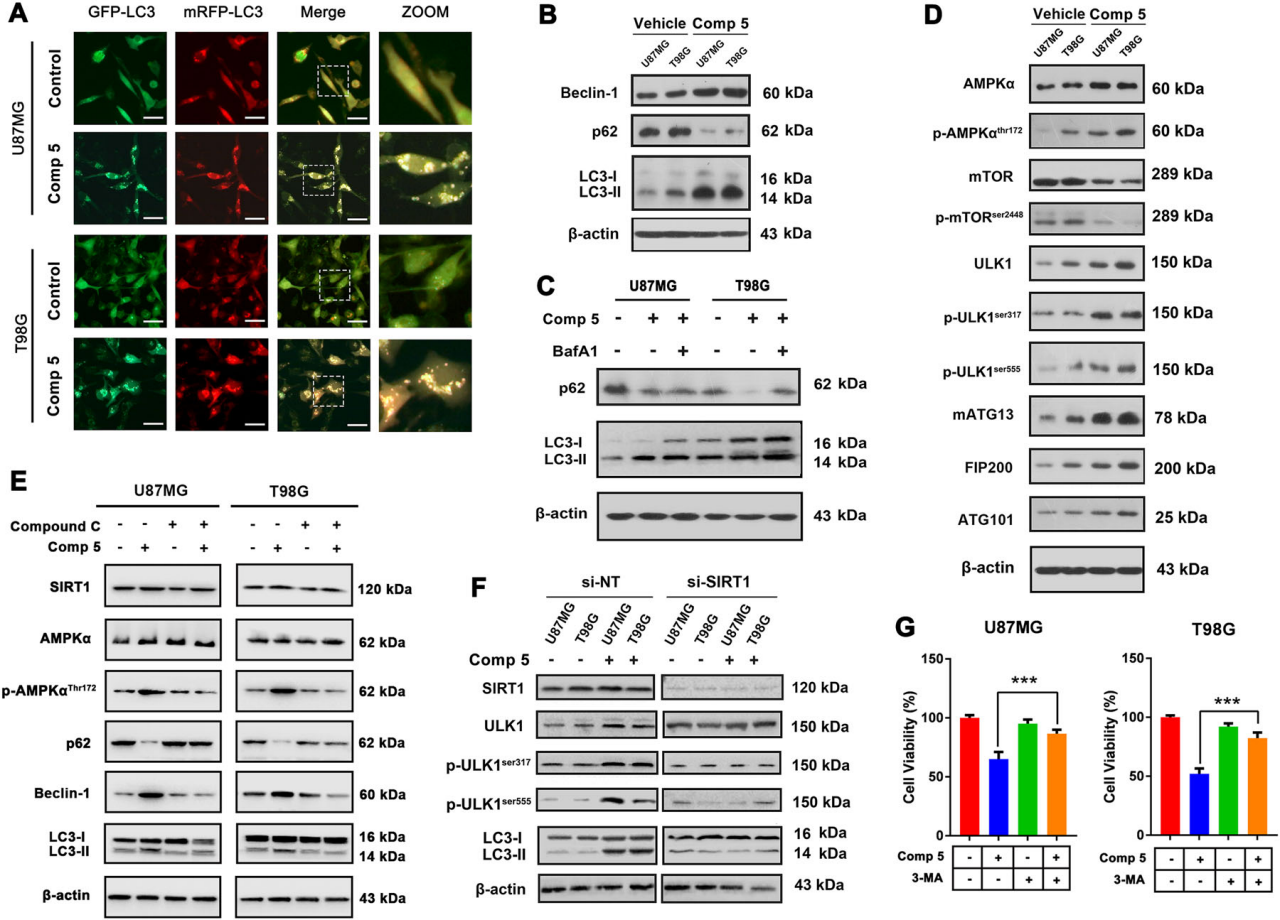
Comp 5 induces autophagy via AMPK-mTOR-ULK complex axis in GBM cells. **a** U87MG and T98G cells were transfected with GFP-mRFP-LC3 plasmid, after co-incubation with 10 μM Comp **5**, the GFP-LC3 puncta were observed by fluorescence microscope. Scale bar = 20 μm. **b** U87MG and T98G cells were treated with 10 μM Comp **5** for 24 h, then the expression levels of Beclin-1, p62, and LC3 were determined by western blot. **c** U87MG and T98G cells were treated with Comp **5** in the presence or absence of BafA1, then the accumulation of p62 and LC3 were detected by western blot. **d** U87MG and T98G cells were treated with 10 μM Comp **5** for 24 h, then the expression levels of AMPKα, p-AMPKα^thr172^, mTOR, p-mTOR^ser2448^, ULK1, p-ULK1^ser317^, p-ULK1^ser555^, mATG13, FIP200, and ATG101 were determined by western blot. **e** U87MG and T98G cells were treated with Comp **5** in the presence or absence of Compound C, then the expression levels of SIRT1, AMPKα, p-AMPKα^thr172^, p62, Beclin-1, and LC3 were detected by western blot. **f** U87MG and T98G cells were transfected with SIRT1 siRNA for indicated time and treated with Comp **5** for additional 24 h. The expression levels of SIRT1, ULK1, p-ULK1^ser317^, p-ULK1^ser555^, and LC3 were determined by western blot. β-actin was used as a loading control. **g** U87MG and T98G cells were treated with 10 μM Comp **5** in the presence or absence of 3-MA (1 mM) for 24 h. Then, cell viabilities were detected by MTT assay. ***P < 0.001 vs. Comp **5**-treated group

To investigate the molecular mechanisms of Comp **5**-induced cell death, we observed the morphologic alternation under phase-contrast microscope, and Comp **5**-treated cells displayed non-apoptotic morphology and massive cytoplasmic vacuoles. Thus, we used GFP-mRFP-LC3 plasmid to examine whether Comp **5** could induce autophagy and observed increasing green fluorescent dots under fluorescence microscope. After GFP-mRFP-LC3 transfection, the cells treated with Comp **5** showed an increase in the number of LC3 puncta (Fig. 3a). To further verify that Comp **5** induces autophagy, we assessed the expression of autophagy markers and found that Comp **5** resulted in increased expression of LC3-II and Beclin-1. Moreover, the level of p62, a selective autophagy substrate, was reduced by treatment with Comp **5** (Fig. 3b). To validate the effect of Comp **5** on autophagy, bafilomycin A1 (BafA1), an inhibitor of autophagosome–lysosome fusion, was used to detect autophagic flux. We found that LC3-II and p62 were obviously accumulated in the presence of BafA1, indicating that autophagic flux is enhanced by treatment with Comp **5** (Fig. 3c). These results collectively suggest that Comp **5** induces autophagy in U87MG and T98G cells.

It is well known that the ULK complex (ULK1-mATG13-FIP200-ATG101) is closely related to autophagosome formation, which is simultaneously regulated by upstream signaling such as AMPK and mTOR under nutrient deprivation condition^16,17^. Thus, we further testify the activation status of the AMPK-mTOR-ULK complex pathway. The phosphorylation of ULK1 at ser317 and ser555, as well as the expression of ULK1, mATG13, FIP200, and ATG101, were remarkably increased after Comp **5** treatment. In addition, the phosphorylation of AMPK was increased, whereas phosphorylation of mTOR was decreased, suggesting that Comp **5** induces autophagy through AMPK-mTOR-ULK complex pathway (Fig. 3d). Subsequently, we used Compound C, an inhibitor of AMPK, to explore whether Comp **5**-induced autophagy is dependent on AMPK activation. We found that Compound C could block the phosphorylation of AMPK and induction of autophagy, which is determined by decreased expression of Beclin-1 and LC3-II, as well as upregulation of p62 (Fig. 3e). To demonstrate that Comp **5**-induced activation of ULK complex is dependent on SIRT1 activation, we used specific siRNA of SIRT1 to silence the expression of SIRT1. Notably, we found that silencing of SIRT1 led to a significant decrease in autophagy induction, as indicated by the decreased phosphorylation of ULK1, and the reduced levels of LC3-II (Fig. 3f). Moreover, we employed autophagy inhibitor 3-MA to examine the role of Comp **5**-induced autophagy in GBM cells. After evaluation of cell viabilities by MTT assay, we found that addition of 3-MA markedly ameliorated cell death induced by Comp **5**, indicating that Comp **5**-induced autophagy promotes cell death in GBM cells (Fig. 3g). Taken together, these results indicate that Comp **5** activates SIRT1 and thus regulating autophagy via the AMPK-mTOR-ULK complex.

### Comp 5 induces mitophagy by SIRT1–PINK1–Parkin pathway

**Fig. 4:**
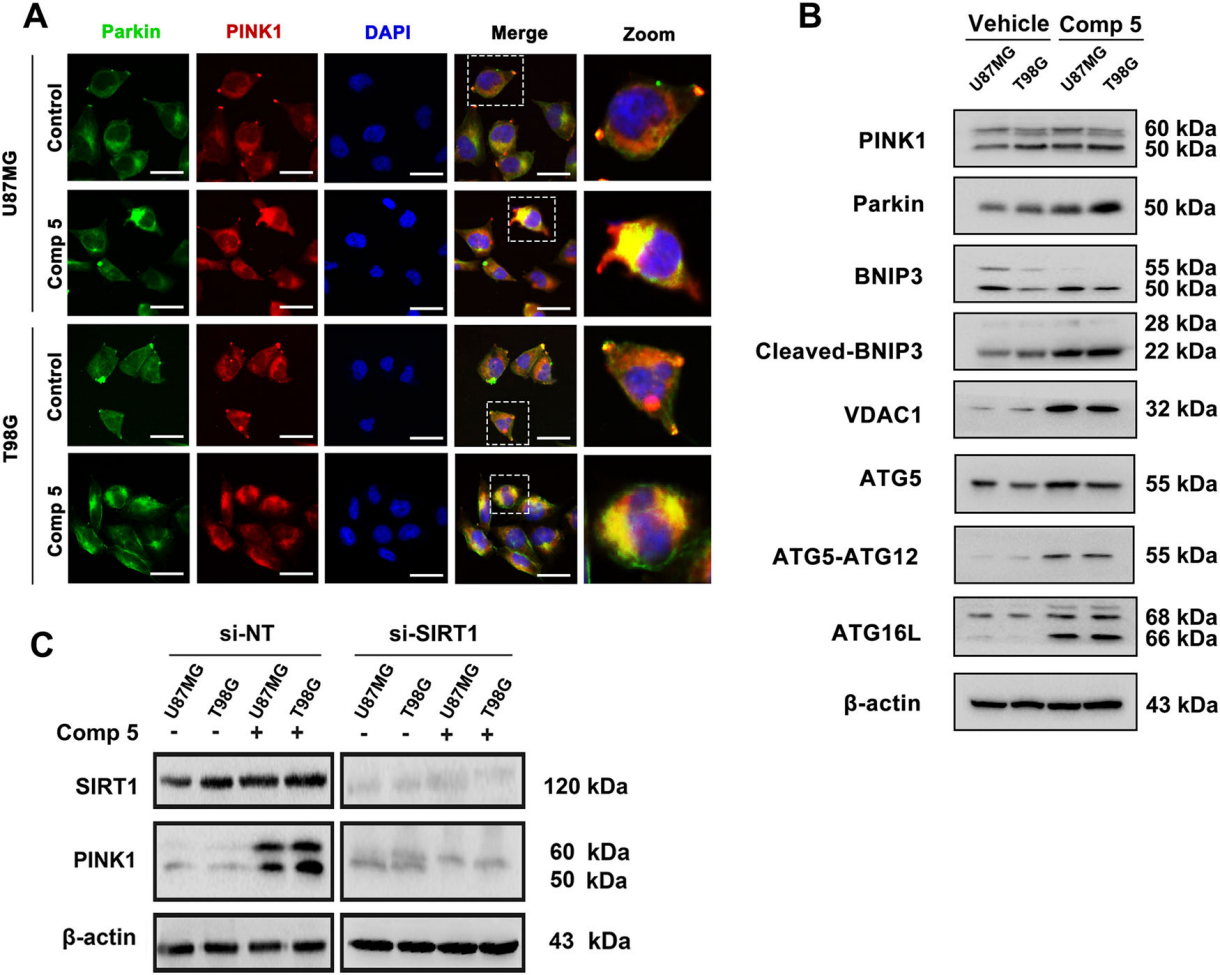
Comp 5 induces mitophagy via SIRT1–PINK1–Parkin pathway in GBM cells. **a** U87MG and T98G cells were treated with 10 μM Comp **5** for 24 h, then fixed with 90% ethanol and incubated with PINK1 primary antibody overnight. After incubated with fluorescence-secondary antibody for 2 h, cells were stained with DAPI for 20 min and observed under fluorescence microscope. Scale bar = 10 μm. **b** U87MG and T98G cells were treated with 10 μM Comp **5** for 24 h, then the expression levels of PINK1, Parkin, BNIP3, VDAC1, ATG5, ATG12-ATG5, and ATG16L were determined by western blot. β-actin was used as a loading control. **c** U87MG and T98G cells were transfected with SIRT1 siRNA for indicated time and treated with or without Comp **5** for additional 24 h. The expression level of PINK1 was determined by western blot. β-actin was used as a loading control

Previous study has revealed that nicotinamide-induced mitophagy is mediated by high NAD+/NADH ratio and SIRT1 protein activation^18^. Thus, we decided to pursue whether Comp **5**induced mitophagy. The PTEN-induced putative kinase 1 (PINK1)-Parkin pathway plays a key role in controlling the process of mitophagy. Particularly, PINK1 accumulates in the mitochondria thereby recruiting Parkin, whereas Parkin ubiquitinases mitochondrial proteins in turn serving as a signal for mitophagy^19,20^. Therefore, we first examined the expression of PINK1 by immunofluorescence staining. PINK1 was obviously expressed in Comp **5**-treated cells (Fig. 4a). To explore the underlying mechanisms of Comp **5**-induced mitophagy, we next examined the expression of mitophagy-related key proteins. We found that Comp **5** markedly increased the expressions of PINK1, Parkin, and VDAC1, as well as the cleavage of BNIP3, a substrate of mitophagy (Fig. 4b). Meanwhile, ATG12-ATG5-ATG16L complex was also involved in Comp **5**-induced mitophagy, which is determined by increased expression of ATG12-ATG5 complex and ATG16L (Fig. 4b). Moreover, knockdown of SIRT1 resulted in inactivation of PINK1, indicating that SIRT1 regulated the activation of PINK1-mediated mitophagy (Fig. 4c). These results demonstrate that Comp **5** induces mitophagy through SIRT1–PINK1–Parkin pathway.

### iTRAQ-based proteomics analyses of Comp 5-induced autophagic/mitophagic mechanisms

**Fig. 5:**
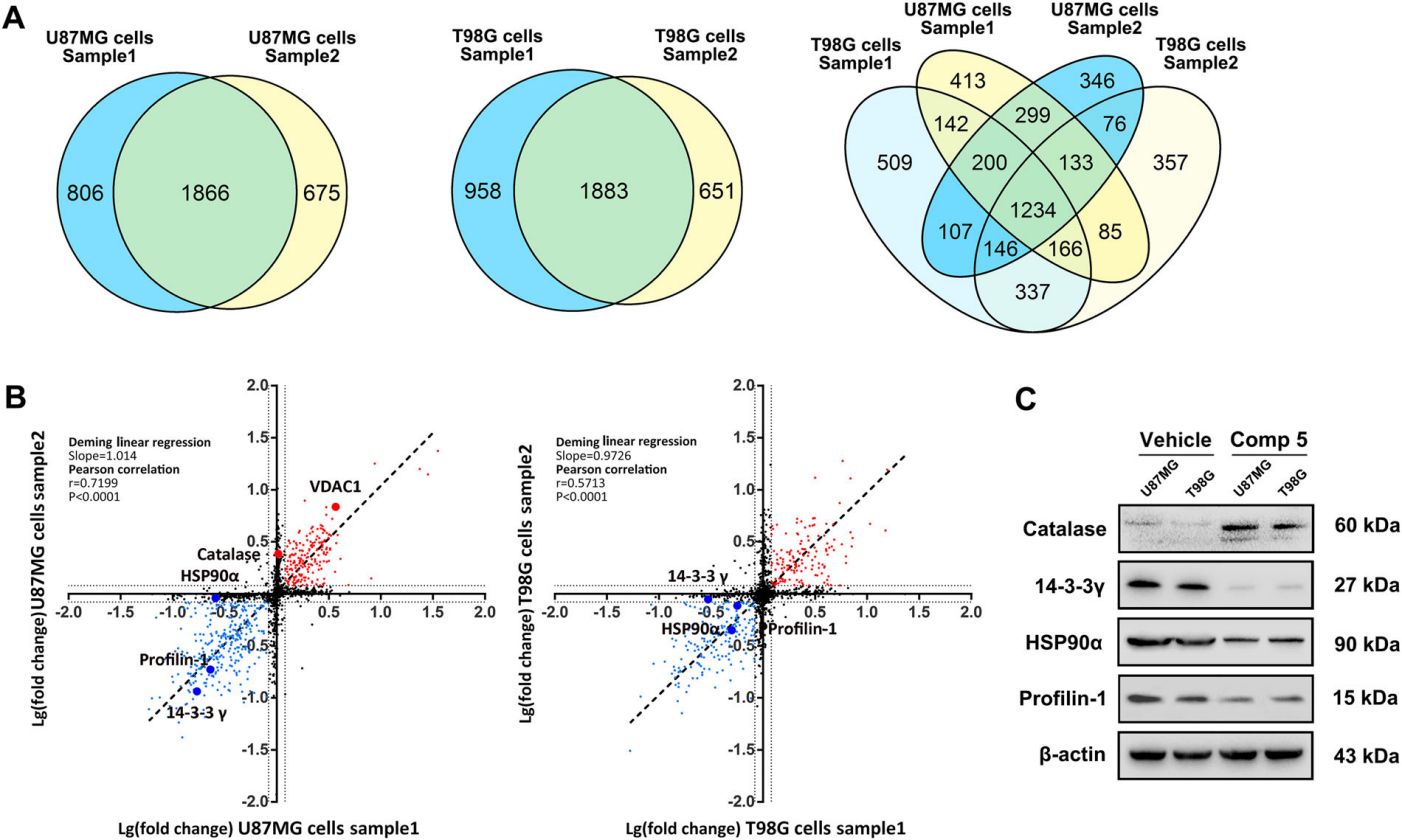
iTRAQ-based proteomics analyses of Comp 5-induced autophagic mechanisms. **a** Venn diagram showed the consensus proteins that were remarkably changed in both U87MG and T98G cells. **b** Correlation of log10 fold changes between the two samples across all changed proteins in both U87MG and T98G cells by Deming linear regression. The proteins that were validated by subsequent experiments were highlighted. **c** The U87MG and T98G cells were treated with 10 μM Comp **5** for 24 h, then the expression levels of catalase, 14-3-3γ, profiling-1, and HSP90α were determined by western blot. β-actin was used as a loading control

To elucidate molecular mechanisms underlying Comp **5**-induced autophagy/mitophagy with SIRT1, iTRAQ-based proteomics analysis was employed to profile differentially expressed proteins in U87MG and T98G cells. We identified five differential expression proteins, 14-3-3γ, catalase, profilin-1, HSP90α, and VDAC1, which were predicted to interact with SIRT1 or to be affected by SIRT1 (Fig. 5a, b). VDAC1 was demonstrated that could be participated in Comp **5**-induced mitophagy (Fig. 5b). Besides above-mentioned finding, we subsequently found that the expression level of catalase was obviously upregulated after treatment of Comp **5**, whereas the expression levels of 14-3-3γ, HSP90α, and profilin-1 were downregulated (Fig. 5c). These results suggest that Comp **5**-induced autophagic cell death is involved with 14-3-3γ, catalase, profilin-1, and HSP90α.

### Comp 5 has a therapeutic potential on GBM by SIRT1-mediated autophagy/mitophagy in vivo

**Fig. 6:**
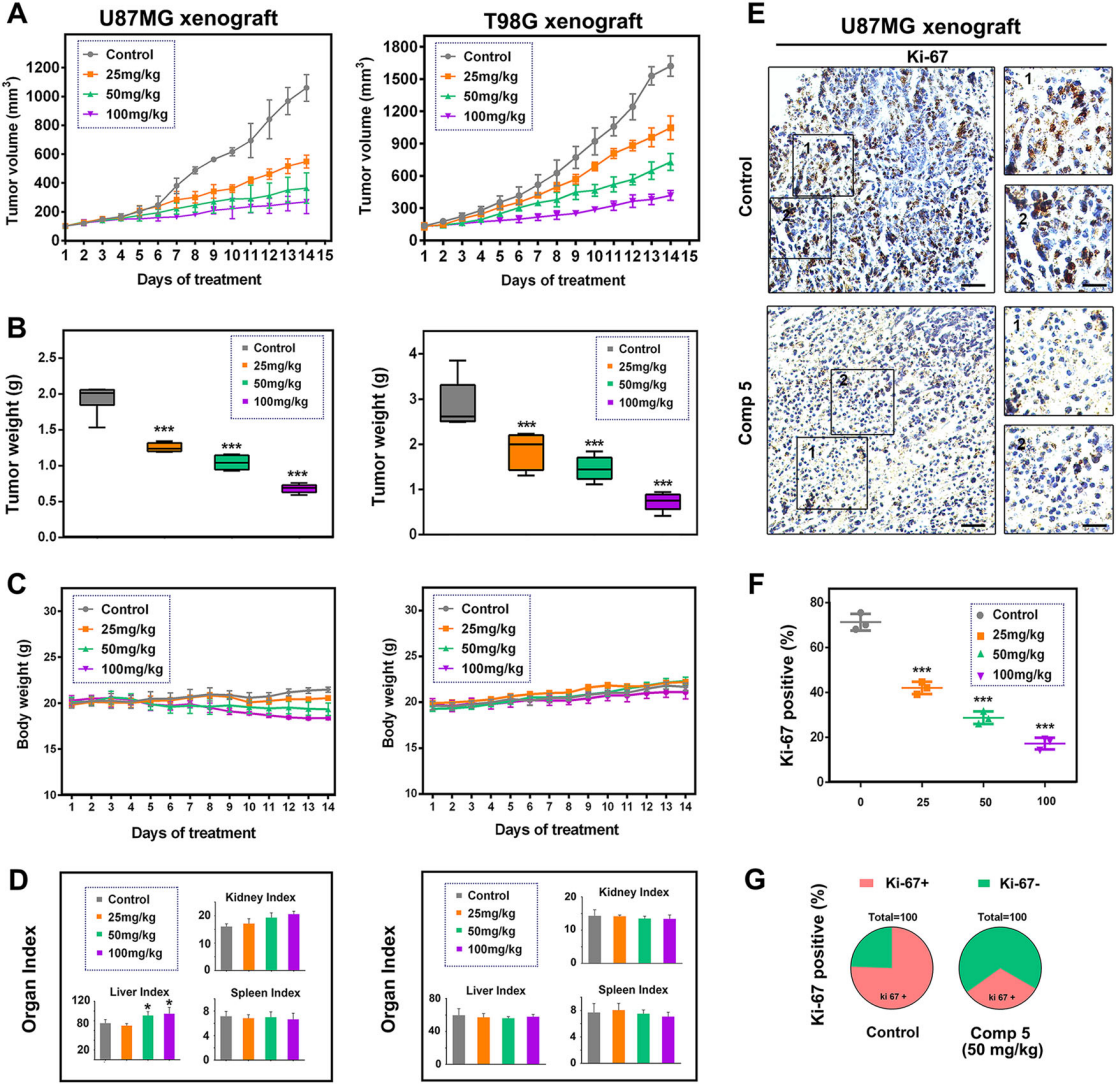
Comp 5 has a therapeutic potential on GBM *in vivo*. **a** Comp **5** obviously decreased U87MG and T98G xenograft tumor volume. Dosage of administration, 25 mg/kg, 50 mg/kg, 100 mg/kg. **b** Representative tumors and the tumor weight change from mice after vehicle and Comp **5** treatment. ***P < 0.001, compared with control group. **c** Body weights of mice during Comp **5** treatment. **d** Organ indexes (kidney, liver, and spleen) of mice were assessed at the end of treatment. *P < 0.05, compared with control group. **e** Immunohistochemical staining of Ki-67. Tumor tissues were excised from the control and median dose group treated U87MG xenograft mice. Scale bar: 200 μm (left panel), 50 μm (right panel) **f**, **g**. Quantitative analysis of Ki-67 positive ratio. ***P < 0.001, compared with control group

**Fig. 7:**
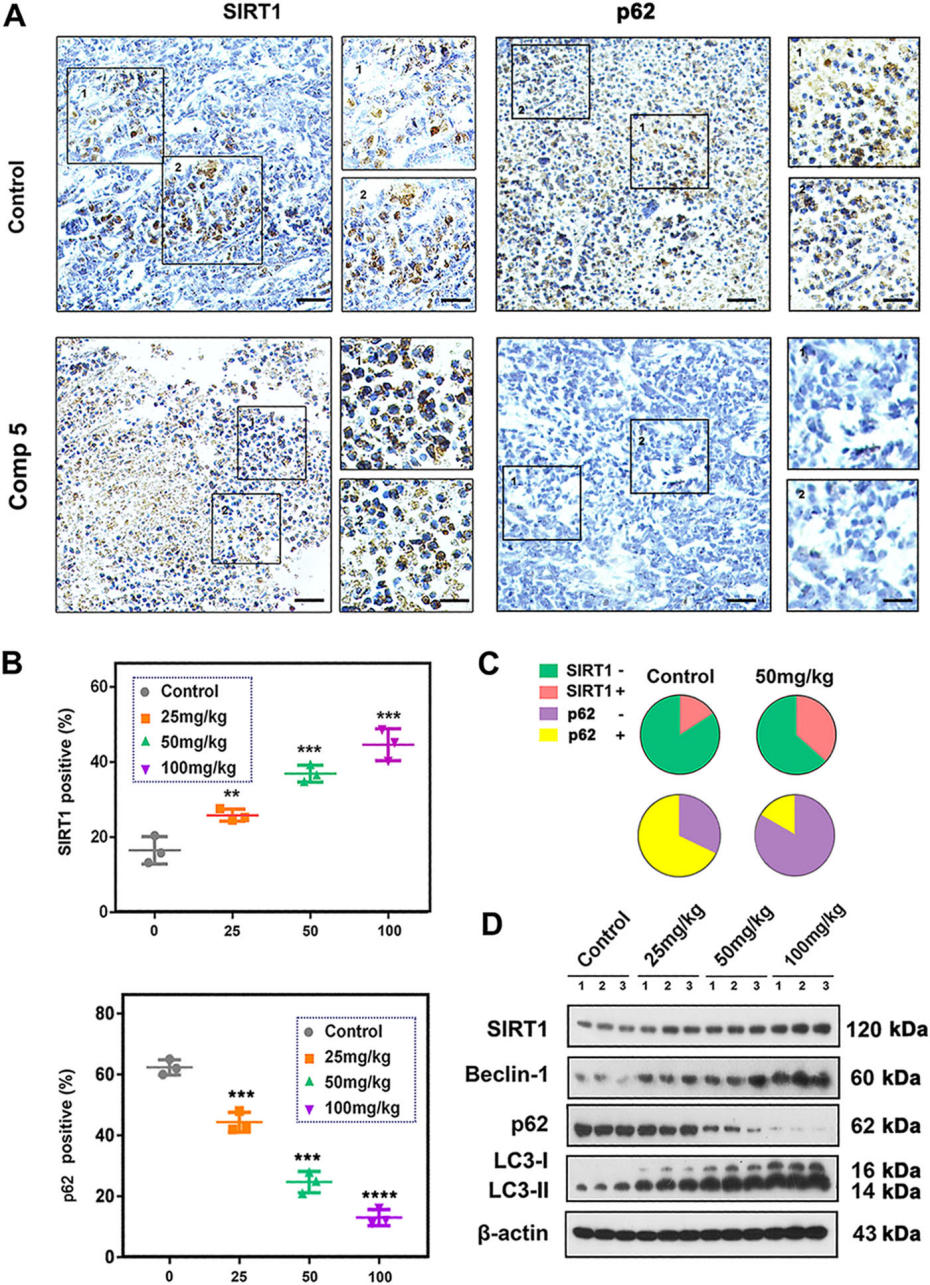
Comp 5 induces autophagy/mitophagy via activating SIRT1 in vivo. **a** Immunohistochemical staining of SIRT1 and p62 tumor tissues were excised from the control and median dose group treated U87MG xenograft mice. **b**, **c** Quantitative analysis of SIRT1 and p62-positive ratios. ***P < 0.001, **P < 0.01, compared with control group. **d** Western blot analysis of SIRT1, Beclin-1, p62, and LC3. Tumor tissues excised from the U87MG xenograft mice were lysed

Based upon the efficacy of Comp **5** on GBM cells in vitro, we proceeded to evaluate its anti-tumor activity in vivo. In the xenograft mouse models of GBM (U87MG and T98G), Comp **5** showed significant inhibition of tumor volume in a dose-dependent manner (Fig. 6a). Similarly, a remarkable loss of tumor weights was observed in all dose groups (*P* < 0.001) (Fig. 6b). Compared with the control group, a slight loss of body weight was induced by high dose of Comp **5** during the 14 days of treatment in U87MG xenograft mice (*P* < 0.01), whereas the loss of body weight in other groups was not obvious (Fig. 6c). By the end of the experiment, the liver and kidney weight indexes of mice were lightly increased in median and high dose groups in U87MG xenograft mice (*P* < 0.01), whereas the spleen weight index was not affected in all dose group, indicating that Comp **5** has no apparent toxicity (Fig. 6d). To test whether Comp **5**-mediated inhibition of tumor growth in vivo was associated with reduced cell proliferation, Ki-67 was analyzed for its immunoreactivity. We found that Comp **5** treatment obviously reduced the number of Ki-67-positive cells in U87MG xenograft mice (Fig. 6e–g). To elucidate the mechanism for the therapeutic efficacy of Comp **5** in vivo, we examined the immunoreactivity of SIRT1 and p62 in tumor tissues of U87MG xenograft mice. As a result, the expression of SIRT1 was increased and p62 was decreased in the Comp **5**-treated tumor tissues (Fig. 7a–c). Next, we carried out western blot analysis to further clarify the mechanism of Comp **5** in vivo. As expected, Comp **5** increased the expression levels of SIRT1, Beclin-1, and LC3-II, as well as the degradation of p62 in vivo, which was in line with the immunohistochemical analysis and the results obtained in vitro (Fig. 7d). Taken together, these results suggest that Comp **5** inhibits tumor growth by SIRT1-mediated autophagy in vivo without obvious toxicity.

## Discussion

Of note, SIRT1 is a class III histone deacetylase that may mediate the protective effects of neurons in neurological diseases such as brain cancer^21,22^. It was reported to be expressed in a high level in healthy organs over the body^5,23^. And the induction of SIRT1 could facilitate treatments of diseases. For instance, it was reported that activating of SIRT1 could significantly attenuate the loss of retinal ganglion cell^24^. And, the spinal cords could be protected by SIRT1 activation against neurological dysfunction^25^. Overexpression of SIRT1 was reported to inhibit cell proliferation and tumor development through the mechanism of downregulation of NF-kB activity and inhibition of cyclin D1 signaling^26^. Besides, activation of SIRT1 was found to cause cellular senescence in a SIRT1-dependent manner^27^. Based on above-mentioned studies, an admirable therapeutic approach may be put forward as targeting activation of SIRT1 for small-molecule drug discovery. In our study, we paid close attention to the potential target for cancer therapies, SIRT1, and focused on searching for a proper compound as a SIRT1-targered activator.

SIRT1 activators can ameliorate the course of cancers or function as inhibitors of cancer cells^28,29^. It was reported that one new dammarane-type triterpenes isolated from stems–leaves of Panax ginseng could activate SIRT1 to inhibit the proliferation of human leukemia HL-60 cells and human hepatocellular carcinoma Hep-G2 cells^30^. A SIRT1 activator YK-3-237 was reported to inhibit the proliferation of triple-negative breast cancer cells^31^. And studies showed that SIRT1 activators could be neuroprotective against optic neuritis induced with a neurotropic strain of mouse hepatitis virus, MHV-A59, or chronic experimental allergic encephalomyelitis induced by immunization with MOG peptide^32,33^. In our study, we used in silico screening, molecular pharmacological technologies and iTRAQ-based proteomics analysis to screen and discover a novel small-molecule activator of SIRT1 (Comp **5**) with brilliant anticancer activity of glioblastoma in vitro and in vivo without obvious toxicity, suggesting that application of SIRT1 activators was a promising strategy in cancer.

Molecular mechanisms of SIRT1 activators reported were multifarious. For instance, YK-3-237 could activate SIRT1 enzyme activities in vitro and deacetylation of mutations in tumor suppressor p53 in a SIRT1-dependent manner^31^. Mechanisms of action of a SIRT1 activator SRT1720 may include, at least in part, the suppression of renal oxidative stress and the TGF-β1/CTGF signaling pathway^34^. In our study, Comp **5**-induced autophagy in U87MG and T98G cells. And the phosphorylation of ULK1 at ser317 and ser555, as well as the expression of ULK1, mATG13, FIP200, and ATG101, were remarkably increased after Comp **5** treatment. Taken together, these results indicate that Comp **5** activated SIRT1 and thus regulating autophagy via the AMPK-mTOR-ULK complex.

Mitophagy is a form of selective autophagy that is specific for the degradation of damaged mitochondria^35,36^. The most studied pathway for mitophagy induction is regulated by PTEN-induced putative kinase 1 (PINK1) and Parkin, where PINK1 recruits Parkin to depolarized mitochondria. When recruited to mitochondria, Parkin ubiquitinates outer mitochondrial membrane proteins for recognition and engulfment by autophagosomes and subsequent degradation in lysosomes. Several Parkin-independent pathways also induce mitophagy^37,38^. In our study, we examined the expression of mitophagy-related key proteins and found that Comp **5** markedly increased the expressions of PINK1, Parkin, and VDAC1. Meanwhile, ATG12-ATG5 complex and ATG16L were increasingly expressed, which were involved in Comp **5**-induced mitophagy. Besides, iTRAQ-based proteomics analysis was employed to profile differentially expressed proteins in U87MG and T98G cells. As a result, we found that the expression level of catalase was obviously upregulated after treatment of Comp **5**, whereas the expression levels of 14-3-3γ, HSP90α, and profilin-1 were downregulated. These results demonstrated that mitophagy was induced by Comp **5** through SIRT1–PINK1–Parkin pathway and was involved with 14-3-3γ, catalase, profilin-1, and HSP90α.

In conclusion, based on the importance of SIRT1 as a vital therapeutic target and the fact that the applications of “SIRT1-modulating autophagy” therapeutic strategy in brain cancer remains in its infancy, we innovatively discovered a novel small-molecule activator of SIRT1 with autophagic/mitophagic mechanisms, which has remarkable anti-tumor activity against glioblastoma in vitro and in vivo. This study will provide a novel approach for cancer therapies, especially glioblastoma, and SIRT1 activators inducing autophagy/mitophagy will be potentially of great value as a novel leading drug for future cancer therapeutics.

## Materials and methods

### Expression profiling in TCGA data set

TCGA brain cancer mRNA gene expression data were downloaded from UCSC Xena at https://xenabrowser.net/. Gene expression profile was measured by using the Illumina HiSeq 2000 RNA Sequencing platform. RSEM (RNA-Seq by Expectation-Maximization) normalized count was used as gene level expression estimates. Low and high-SIRT1 expression was determined by an obvious expression level jump.

### TMA analysis

The glioblastoma tissue microarrays (GL805a, GL805b) and normal brain tissue microarray (BNC17011a) were obtained from Cybrdi, Inc. The expression of SIRT1 was evaluated by immunohistochemistry using anti-SIRT1 antibody (1:200) as previously described^39^. The microarray contained glioblastoma tumor tissues from 70 individuals plus 35 normal brain tissues. The immunostaining intensity was indicated by four grades (0, negative; 1, weakly positive; 2, positive; 3, strongly positive) and the percentage of cell staining at each intensity level was graded as the proportion of stain-positive cells and divided into five grades 0 (<5%), 1 (5–25%), 2 (26–50%), 3 (51–75%), 4 (>75%). The final score was calculated as intensity score × the proportion of stain-positive cells.

### In silico screening of SIRT1 activators

The pharmacophore model was constructed from the X-ray crystal structure of SIRT1 (PDB code: 4ZZJ) based on Leu206, Pro211, Gln222, Ile227 residues by Accelrys Discovery Studio 3.5 (SanDiego, CA, USA). The Libdock screening was performed as the standard protocol, the max hits to save parameters was set 2 and the other parameters were default values^40^. The CDOCKER protocol was employed as further docking approach to conduct semi-flexible docking to re-rank the top 20 small-molecule compounds^41^.

### In vitro SIRT1 deacetylase activity assay

The in vitro SIRT1 deacetylase activity was determined using a SIRT1 Activity Assay Kit (Abcam, ab156065) following the manufacturer’s protocol^42^. In brief, Fluoro-Substrate Peptide, Fluoro-Deacetylated Peptide and NAD were successively added to SIRT1 assay buffer followed by incubation with the tested compounds, as well as the developer buffer and recombinant SIRT1 (5 mg) at 37 °C for 60 min. After excitation at 350 nm, emitted light was detected at 460 nm using microtiter plate fluorometer. The fluorescence intensity of the assay buffer was subtracted from each experimental sample.

### RNA isolation and quantitative PCR

Total RNA was isolated using TRIzol reagent (Life Technologies) and then reverse-transcribed using iScript Cdna Synthesis Kit (Bio-Rad). The resulting cDNA was used for qPCR using iTaq Universal SYBR Green Supermix (Bio-Rad) with gene-specific primers and the results were normalized to β-actin control.

In reverse transcription PCR (RT–PCR) assays, the primer sequences for *SIRT1* are 5′-GCAGATTAGTAGGCGGCTTG-3′ and 5′-TCTGGCATGTCCCACTATCA-3′, and for *ACTB* are: 5′-CATGTACGTTGCTATCCAGGC-3′ and 5′-CTCCTTAATGTCACGCACGAT-3′.

### Cellular SIRT1 activity assay

The celluar SIRT1 activity in an immunoprecipitate was performed using SIRT1 Activity Assay Kit Fluorometric (ab156065) according to the manufacturer’s instructions. Treat cells by adding fresh media containing test compound for desired time. To harvest cells under non-denaturing conditions, remove media and rinse cells once with ice-cold phosphate-buffered saline (PBS). Remove PBS and add 0.5 mL 1Xice-cold Cell Lysis Buffer to each plate (10 cm dish) and incubate the plate on ice for 5 min. Scrape cells off the plate and transfer to microcentrifuge tubes. Sonicate four times for 5 s each on ice. Microcentrifuge for 10 min at 4 °C, and transfer the supernatant to a new tube to obtain the cell lysate. Take 200 μL cell lysate and incubate with a suitable anti-SIRT1 antibody or anti-IgG according to the manufacturer’s instructions. Add Protein A agarose beads (20 μL of 50% bead slurry). Incubate with gentle rocking for 1–3 h at 4 °C. Microcentrifuge for 30 s at 4 °C. Wash pellet three times with 500 μL of 1X Cell Lysis Buffer and once with 500 μL of SIRT1 assay buffer (50 mM Tris-HCl (pH 8.8), 0.5 mM dithiothreitol). After immunoprecipitation, add reaction mixture containing Fluoro-Substrate peptide solution to Protein A agarose beads as an “Enzyme Sample” and measure NAD-dependent deacetylase activity.

### SIRT3 and SIRT6 activity assay

The SIRT3 and SIRT6 activity assay were performed using SIRT3 Activity Assay Kit (Enzo Life Sciences, BML-AK557) and SIRT6 Activity Assay Kit (abcam, ab156068) according to the manufacturer’s instructions. In brief, Fluoro-Substrate Peptide, Fluoro-Deacetylated Peptide and NAD were successively added to SIRT3 and SIRT6 assay buffer followed by incubation with the tested compounds, as well as the recombinant SIRT (5 mg) at 37 °C for 45 min. For SIRT3 assay, stop the assay with 1× Developer II/2 Mm nicotinamide, excitation at 360 nm, emitted light was detected at 460 nm using microtiter plate fluorometer. For SIRT6 assay, excitation at 480 nm, emitted light was detected at 530 nm using microtiter plate fluorometer. The fluorescence intensity of the assay buffer was subtracted from each experimental sample.

### Cell culture and reagents

U87MG and T98G cells were purchased from ATCC. Cells were cultured in Dulbecco’s Modified Eagle Medium with 10% fetal bovine serum and incubated with 5% CO_2_. All candidate compounds tested in this study were purchased from Life Chemicals Inc (Kyiv, Ukraine). MTT (M2128), 3-MA (M9281), and DAPI (D9542) were purchased from Sigma-Aldrich (St. Louis, MO, USA). MitoTracker Red was purchased from Thermo Fisher Scientific Inc. (NY, USA). Bafilomycin A1 (ab120497) and Resveratrol (ab120726) were purchased from Abcam (Cambridge, UK). Antibodies used in this study were as follow: SIRT1 (2496, CST, MA, USA), Acetyl-Histone H3 (9649, CST), Histone H3 (4499, CST), p53 (2527, CST), Acetyl-p53 (2525, CST), AMPK (5831, CST), p-AMPK^thr172^ (2535, CST), ULK1 (8054, CST, MA, USA), p-ULK1^ser317^ (12753, CST), p-ULK1^ser555^ (12753, CST), mAtg13 (13273, CST), Atg101 (13492, CST), FIP200 (12436, CST), mTOR (2983, CST), p-mTOR^ser2448^ (12436, CST), Beclin-1 (3495, CST), LC3B (3868, CST), SQSTM1/p62 (5114, CST), BNIP3 (44060, CST), PINK1 (6946, CST), Parkin (4211, CST), VDAC1 (4661, CST), ATG5 (12594, CST), ATG12 (4180, CST), ATG16L (8089, CST), 14-3-3γ (5522, CST), catalase (12980, CST), Profilin-1 (3237, CST), HSP90 (4877, CST), β-actin (66009-1-Ig, Proteintech, IL, USA).

### Plasmid and siRNA transfections

For plasmid transfection, cells were transfected with GFP-mRFP-LC3 (HB-AP210 0001, HANBIO, China) for indicated times and observed under a fluorescence microscopy (DM2500, LEICA). For siRNA transfection, cells were transfected with SIRT1 or negative control siRNAs at 100 nM final concentration using Lipofectamine RNAiMAX reagent (Invitrogen) according to the manufacturer’s instructions. The transfected cells were used for subsequent experiments 36 h later.

### Immunofluorescence assay

The immunofluorescence assay was performed as previously described^39^. In brief, nonspecific antibody binding was blocked by incubating with PBS containing 1.5% goat serum. Cells were sequentially incubated, starting with primary antibodies (PINK1, 1:500; Parkin, 1:1000) diluted in PBS containing 1% bovine serum albumin (BSA) incubated overnight at 4 °C, followed by addition of fluorescence-labeled secondary antibodies (TRITC, ab6718; FITC, ab6717) for 1 h at room temperature. Images were captured by fluorescence microscope (DM2500, LEICA).

### Western blot analysis

Cells were treated with Comp **5** for indicated times. Both adherent and floating cells were collected, then the cell pellets were resuspended with lysis buffer consisting of Hepes 50 mM pH 7.4, Triton-X-100 1%, sodium orthovanada 2 mM, sodium fluoride 100 mM, edetic acid 1 mM, phenylmethane sulfonyl fluoride 1 mM, aprotinin (Sigma, MO, USA) 10 mg/L and leupeptin (Sigma) 10 mg/L and lysed at 4 °C for 1 h. After 12,000 rpm centrifugation for 15 min, the protein content of supernatant was determined by the Bio-Rad DC protein assay (Bio-Rad Laboratories, Hercules, CA, USA). Equal amounts of the total protein were separated by 10–15% sodium dodecyl sulfate polyacrylamide gel electrophoresis and transferred to polyvinylidene difluoride membranes, the membranes were soaked in blocking buffer (5% skimmed milk or BSA). Proteins were detected using primary antibodies, followed by horseradish peroxidase (HRP)-conjugated secondary antibody and visualized by using ECL as the HRP substrate.

### iTRAQ-based proteomics analysis

Two separate iTRAQ-based proteomics analysis were performed in U87MG and T98G cells. In brief, cells were dissolved in lysis buffer and labeled with iTRAQ-labeling reagents. After 2D LC analysis and tandem mass spectrometry analysis, protein identification and relative iTRAQ quantification were performed with ProteinPilot™ Software 4.2 (AB SCIEX) using the Paragon™ algorithm for the peptide identification. Results with iTRAQ ratio cutoff values of 1.2 and 0.8 for fold-change and number cutoff values of three for quantifiable peptides for in protein abundance were accepted.

### Tumor xenograft model

All experiments protocols used in this study were carried out in accordance with guidelines of the animal ethics committee (Southern Medical University). Forty-eight female nude mice (BALB/c, 6–8 weeks, 20–22 g) were injected subcutaneously with U87MG or T98G cells (5 × 10^6^ cells/mouse). When the tumors reached 100 mm^3^ in volume (*V* = *L* × *W*^2^/2), the mice were divided into four groups, respectively. Three groups were treated with different doses of Comp **5** (25 mg/kg; 50 mg/kg; 100 mg/kg) once a day by intraperitoneal injection for 14 days, whereas the control group was treated with vehicle control (5% CMC-Na). During the treatment, the tumor volumes and body weight were measured every day until the end of the study. At the end of treatment, all mice were killed. The spleen, liver, kidney, and tumor tissue were harvested, weighed, and photographed. Then, the tumor tissues were frozen in liquid nitrogen or fixed in formalin immediately.

### Immunohistochemistry

Sections of the tumor were submerged into ethylenediaminetetraacetic acid antigenic retrieval buffer (pH 8.0) or citrate buffer (pH 6.0), and microwaved for antigenic retrieval. Then the slides were incubated with rabbit anti-Ki-67 antibody (1:400), or anti-SIRT1 antibody (1:400), or anti-p62 antibody (1:200) for 30–40 min at 37 °C. Normal rabbit/mouse IgG was used as a negative control. The slides were then treated by HRP polymer conjugated secondary antibody for 30 min and developed with diamino-benzidine solution. Meyer’s hematoxylin was used as a counterstain.

### Statistical analysis

All the presented data and results were confirmed by at least three independent experiments. The data are expressed as means ± SEM and analyzed with GraphPad Prism 6.0 software. Statistical comparisons were made by one-way analysis of variance and Student’s *t* test. *P* < 0.05 was considered statistically significant.
